# Omadacycline in the treatment of community-acquired bacterial pneumonia in patients with comorbidities: a *post-hoc* analysis of the phase 3 OPTIC trial

**DOI:** 10.3389/fmed.2023.1225710

**Published:** 2023-07-28

**Authors:** George D. Rodriguez, Nathan Warren, Roman Yashayev, Surya Chitra, Maria Amodio-Groton, Kelly Wright

**Affiliations:** ^1^Division of Antimicrobial Stewardship, New York-Presbyterian Queens, Flushing, NY, United States; ^2^Columbia University School of Nursing, New York, NY, United States; ^3^Paratek Pharmaceuticals, Inc., King of Prussia, PA, United States

**Keywords:** community-acquired bacterial pneumonia, omadacycline, fluoroquinolones, antibiotic resistance, oral antibiotics

## Abstract

**Introduction:**

The 2019 American Thoracic Society/Infectious Disease Society of America guidelines recommend respiratory fluoroquinolones to treat community-acquired bacterial pneumonia (CABP) in adults with comorbidities. Fluoroquinolones are effective against both typical and atypical pathogens. However, fluoroquinolone treatment has a risk of adverse effects, and the Food and Drug Administration has issued black box safety warnings for their use. Inpatient use of fluoroquinolones has reduced as a result; however, most antibiotic courses are completed as outpatients and discharge prescriptions account for the majority of fluoroquinolone use. As such, a new treatment option is needed to replace fluoroquinolones. Omadacycline is an aminomethylcycline antibiotic with a broad spectrum of activity and is available as a once-daily intravenous or bioequivalent oral formulation.

**Methods:**

This study assessed the safety and clinical efficacy of omadacycline compared with moxifloxacin for the treatment of adult CABP patients with Pneumonia Severity Index (PSI) risk class II/III and ≥1 comorbidity through a *post-hoc* analysis of the phase 3 OPTIC study (NCT02531438).

**Results:**

In total, 239 omadacycline- and 222 moxifloxacin-treated patients were assessed. The median age was similar between groups (omadacycline: 57 years; moxifloxacin: 58 years), with 26.0% and 26.6%, respectively, ≥65 years of age. Early clinical response was 91.6% for patients with ≥1 comorbidity treated with omadacycline and 91.4% for those treated with moxifloxacin. Post-treatment evaluation results for overall response were 89.1% in the omadacycline group and 87.4% in the moxifloxacin group.

**Conclusion:**

Safety warnings have reduced inpatient use of fluoroquinolones; however, outpatient and discharge prescriptions account for the majority of fluoroquinolone use. Outpatients with comorbidities need an efficacious alternative to fluoroquinolones. Omadacycline maintains the similar efficacy and benefits of fluoroquinolones as a once-daily, monotherapy, bioequivalent oral option with potent *in vitro* activity against the most common CABP pathogens, including *S. pneumoniae* and atypical pathogens, but offers a materially different safety profile consistent with its tetracycline heritage. In conclusion, both omadacycline and moxifloxacin exhibited similar efficacy in patients with PSI risk class II/III and comorbidities. Omadacycline fulfills an unmet need as an oral monotherapy treatment option for adult patients with CABP, which will further reduce the use of fluoroquinolones.

**Clinical trial registration:**

https://www.clinicaltrials.gov/study/NCT02531438, identifer: NCT02531438; https://www.clinicaltrialsregister.eu/ctr-search/search?query=2013-004071-13, identifier: EudraCT #2013-004071-13.

## 1. Introduction

For the treatment of community-acquired bacterial pneumonia (CABP) in adults with comorbidities, the 2019 American Thoracic Society (ATS)/Infectious Diseases Society of America (IDSA) guidelines recommend either a combination therapy of amoxicillin/clavulanate or cephalosporin plus macrolide or doxycycline or monotherapy with a respiratory fluroquinolone (1). Fluoroquinolones are recommended as monotherapy for treating CABP as they are effective against the most common bacterial pathogens, both typical and atypical (1). However, fluoroquinolone treatment has a risk of adverse effects (AE). The most commonly identified AE is tendinopathy, with other reported AE domains including muscular, cognitive, psychiatric, and gastroenterological AEs (2). These AEs may become severe and require the discontinuation of fluoroquinolone treatment. *Streptococcus pneumoniae* is the most prevalent cause of CABP (3) and has become increasingly resistant to oral antibiotics, such as macrolides, penicillin, cephalosporins, and doxycycline (4–6). The Centers for Disease Control and Prevention (CDC) have classified drug-resistant *Streptococcus pneumoniae* as a serious public threat (7). In the United States, pneumonia and influenza are the ninth leading cause of death (8). Globally, lower respiratory infections, such as pneumonia, are the leading cause of death from communicable diseases (9). Alternative treatments are required to address the high unmet need, particularly concerning oral once-daily, fluoroquinolone-sparing, monotherapy antibiotic options.

Omadacycline is an aminomethylcycline antibiotic with *in vitro* activity against a broad spectrum of pathogens, including drug-resistant *Streptococcus pneumoniae*, β-lactamase-positive *Haemophilus influenzae*, and atypical pathogens such as *Legionella pneumophila* (10). Owing to modifications to the core tetracycline structure, omadacycline maintains antibacterial activity against pathogens expressing bacterial ribosomal protection proteins and tetracycline-specific efflux pumps, which are the two most common tetracycline resistance mechanisms (11). Omadacycline is available as a once-daily intravenous (IV) or bioequivalent oral formulation (12). The phase 3 OPTIC (Omadacycline for Pneumonia Treatment in the Community; NCT02531438) study demonstrated similar clinical efficacy between omadacycline and moxifloxacin, a respiratory fluoroquinolone, in adult patients with CABP (13). OPTIC enrolled patients with various degrees of disease severity and comorbidities. This allowed further analysis of outcomes for a subset of patients for whom outpatient treatment could be considered when applying the preferential, validated prediction rule for prognosis; the Pneumonia Severity Index (PSI); and who would require combination therapy with amoxicillin/clavulanate or cephalosporin AND macrolide or doxycycline OR monotherapy with respiratory fluoroquinolone if treated according to the ATS/IDSA community-acquired pneumonia (CAP) treatment guidelines (1). The ATS/IDSA guidelines continue to recommend respiratory fluoroquinolones for the management of CAP, despite black box safety warnings. This is due to their established efficacy in CAP, low resistance rates, broad coverage of pathogens associated with CAP, including typical and atypical pathogens, high bioavailability, and the convenience of monotherapy (1). Omadacycline shares these beneficial attributes with fluoroquinolones; however, the tetracycline class, including omadacycline, has a materially different safety profile, as evidenced by the FDA warnings listed in the prescribing information (14). The objective of this study was to determine the safety and clinical efficacy of omadacycline compared with moxifloxacin for the treatment of adult patients with CABP and comorbidities who were eligible for treatment as outpatients in the phase 3 OPTIC study.

## 2. Methods

**Figure 1:**
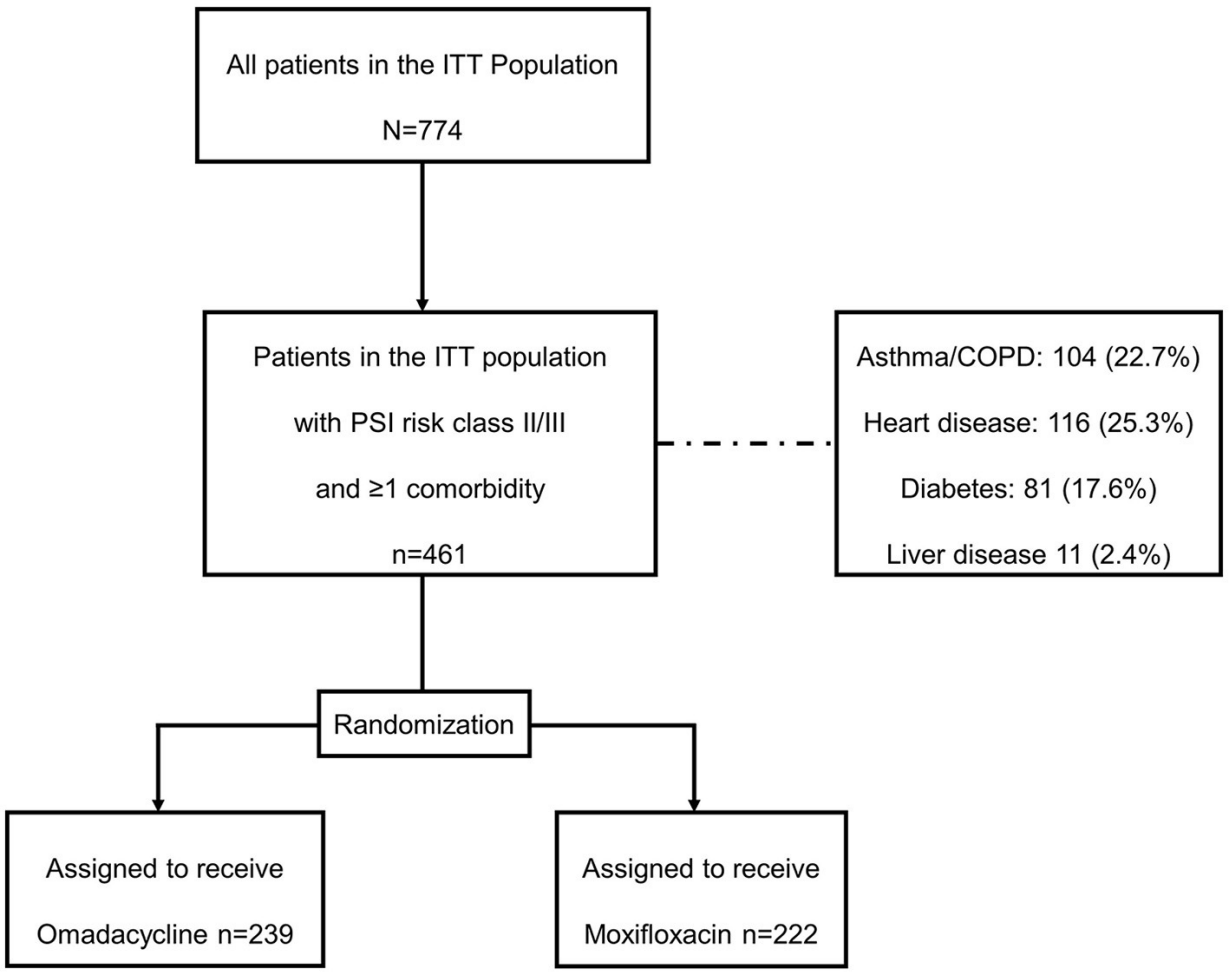
Patient disposition. COPD, chronic obstructive pulmonary disease; ITT, intention-to-treat; PSI, Pneumonia Severity Index.

In the OPTIC study (NCT02531438), patients were eligible for inclusion if they had at least three of the following four CABP symptoms: cough, dyspnea, pleuritic chest pain, or purulent sputum; two or more abnormal vital signs, with at least one clinical sign or laboratory finding associated with CABP, radiologically confirmed pneumonia, and be characterized as Pneumonia Severity Index (PSI) risk class II, III, or IV (13). This *post-hoc* analysis was performed for all patients with PSI risk classes II or III and at least one comorbidity (asthma/chronic obstructive pulmonary disease [COPD], diabetes mellitus, heart disease, liver disease, and renal disease), and outcomes were further described for each comorbidity. Patient disposition and randomization are shown in Figure 1. Adult patients aged 18 years or older were randomly assigned 1:1 to receive 7 to 14 days of either omadacycline (two doses of 100 mg every 12 h administered IV, then every 24 h, with the option to transition to 300 mg every 24 h, taken orally after at least 3 days) or moxifloxacin (400 mg every 24 h administered IV, with the option to transition to 400 mg every 24 h, taken orally after at least 3 days).

The primary efficacy outcome was an early clinical response (ECR), defined in the OPTIC study as clinical success, assessed at 72–120 h after the first dose of a trial drug in the intention-to-treat (ITT) population (13). Clinical success was defined as survival with improvement of at least one level compared with baseline (e.g., from moderate to mild) in at least two CABP symptoms with no worsening in other CABP symptoms. A key secondary endpoint was the investigator-assessed clinical response at the post-treatment evaluation (PTE) performed 5–10 days after the last dose of the trial drug, which was defined as survival with resolution or improvement in the signs and symptoms of infection to the extent that further antibacterial therapy was not necessary (13).

## 3. Results

### 3.1. Patient demographics

Baseline demographics and clinical characteristics for the 239 omadacycline- and 222 moxifloxacin-treated patients who were included in this analysis are shown in Table 1. Demographics between both groups were generally similar, with a median age of 57 years for the omadacycline group and 58 years for the moxifloxacin group, with 26.0% and 26.6%, respectively, 65 years of age or older. The mean body weight was similar between the two groups (79 kg in the omadacycline group and 80 kg in the moxifloxacin group), and each group had an even split of patients in the three BMI categories of under 25 kg/m^2^, 25–30 kg/m^2^ and >30 kg/m^2^, respectively. The majority of patients had normal renal function (74% of the omadacycline group and 77% of the moxifloxacin group, with 11% and 9% with moderate impairment and 16% and 15% with mild impairment). Approximately half of each group had a medical history of hypertension, and around one-quarter had asthma, COPD, or heart disease. One-fifth of patients had diabetes; liver disease was observed in 2% of the omadacycline group and 3% of the moxifloxacin group.

**Table 1:** Baseline demographics and clinical characteristics in patients with a Pneumonia —Severity Index^a^ risk class of II or III and ≥1 comorbidity, ITT population^b^.

**Characteristic**	**Omadacycline (*N* = 239)**	**Moxifloxacin (*N* = 222)**
**Median age (range), years**	**57 (19–85)**	**58 (19–86)**
**Age group**, ***n*** **(%)**		
18–65 years	177 (74.0)	163 (73.4)
>65–75 years	41 (17.2)	40 (18.0)
>75 years	21 (8.8)	19 (8.6)
**Sex**, ***n*** **(%)**		
Female	115 (48.1)	106 (47.7)
Male	124 (51.9)	116 (52.3)
**Race**, ***n*** **(%)**		
White	223 (93.3)	201 (90.5)
Non-white	16 (6.7)	21 (9.5)
**Geographic region**, ***n*** **(%)**		
United States	1 (0.4)	0
Rest of World	238 (99.6)	222 (100.0)
**Mean weight (SD), kg**	**78.8 (18.8)**	**79.8 (18.6)**
**BMI**, ***n*** **(%)**		
<25 kg/m^2^	89 (37.2)	73 (32.9)
25–30 kg/m^2^	78 (32.7)	76 (34.2)
>30 kg/m^2^	72 (30.1)	73 (32.9)
**Renal function**, ***n*** **(%)**		
Moderate impairment (CrCl <60 mL/min)	26 (10.9)	19 (8.6)
Mild impairment (CrCl 60 to 89 mL/min)	37 (15.5)	33 (14.9)
Normal (CrCl >89 mL/min)	176 (73.6)	170 (76.5)
**Select past medical history**, ***n*** **(%)**		
Hypertension	107 (44.8)	103 (46.4)
Asthma/COPD^c^	60 (25.1)	44 (19.8)
Heart disease^d^	57 (23.8)	59 (26.6)
Diabetes mellitus	41 (17.2)	40 (18.0)
Liver disease^e^	5 (2.1)	6 (2.7)

BMI, body mass index; COPD, chronic obstructive pulmonary disease; CrCl, creatine clearance; ITT, intention-to-treat; PSI, Pneumonia Severity Index; SD, standard deviation.

a PSI scores allowed the placement of patients with pneumonia into five risk classes, where higher risk classes indicated greater risk of death; in this subgroup analysis, only patients in risk class II (PSI score, 51 to 70) and III (71 to 90) were included.

b The ITT population included all patients who underwent randomization. There were no significant between-group differences (P<0.05) calculated with the use of Fisher's exact test (for categorical variables) or the Wilcoxon rank-sum test (for continuous variables). Percentages may not total 100 because of rounding.

c Includes patients with symptomatic asthma with wheezing and/or mild-to-moderate COPD. Patients with severe COPD were excluded.

d Heart disease was defined as coronary artery disease, cardiomyopathy, hypertensive heart disease, left ventricular failure, left ventricular hypertrophy, or myocardial fibrosis.

e Liver disease was defined as any hepatitis B, hepatitis C, hepatic steatosis, alcoholic liver disease, hepatic cirrhosis, non-alcoholic steatohepatitis, or hepatic failure.

### 3.2. Early clinical response

Overall, ECR was 91.6% for patients with at least one comorbidity treated with omadacycline and 91.4% for moxifloxacin-treated patients (Table 2). Similarly, clinical response rates observed in patients with at least two comorbidities remained high, at 94.6% for omadacycline and 94.5% for moxifloxacin.

**Table 2:** Clinical response at early clinical response and post-treatment evaluation in Pneumonia Severity Index risk class II/III patients by number of comorbidities, ITT population.

	**Patients with** ≥**1 comorbidity**			**Patients with** ≥**2 comorbidities^a^**		
**Efficacy outcome**	**Omadacycline** ***n*** **(%)**	**Moxifloxacin** ***n*** **(%)**	**Difference (95% CI)**	**Omadacycline** ***n*** **(%)**	**Moxifloxacin** ***n*** **(%)**	**Difference (95% CI)**
ITT population, N	239	222		74	73	
**Early clinical response**						
Clinical success	219 (91.6)	203 (91.4)	0.2 (−5.0, 5.5)	70 (94.6)	69 (94.5)	0.1 (−8.4, 8.6)
Clinical failure or indeterminate	20 (8.4)	19 (8.6)		4 (5.4)	4 (5.5)	
Clinical failure	15 (6.3)	15 (6.8)		4 (5.4)	3 (4.1)	
Indeterminate	5 (2.1)	4 (1.8)		0	1 (1.4)	

CI, confidence interval; ITT, intention-to-treat.

a Patients with ≥2 comorbidities are a subset of patients from the patients with ≥1 comorbidity columns.

### 3.3. Post-treatment evaluation

**Figure 2:**
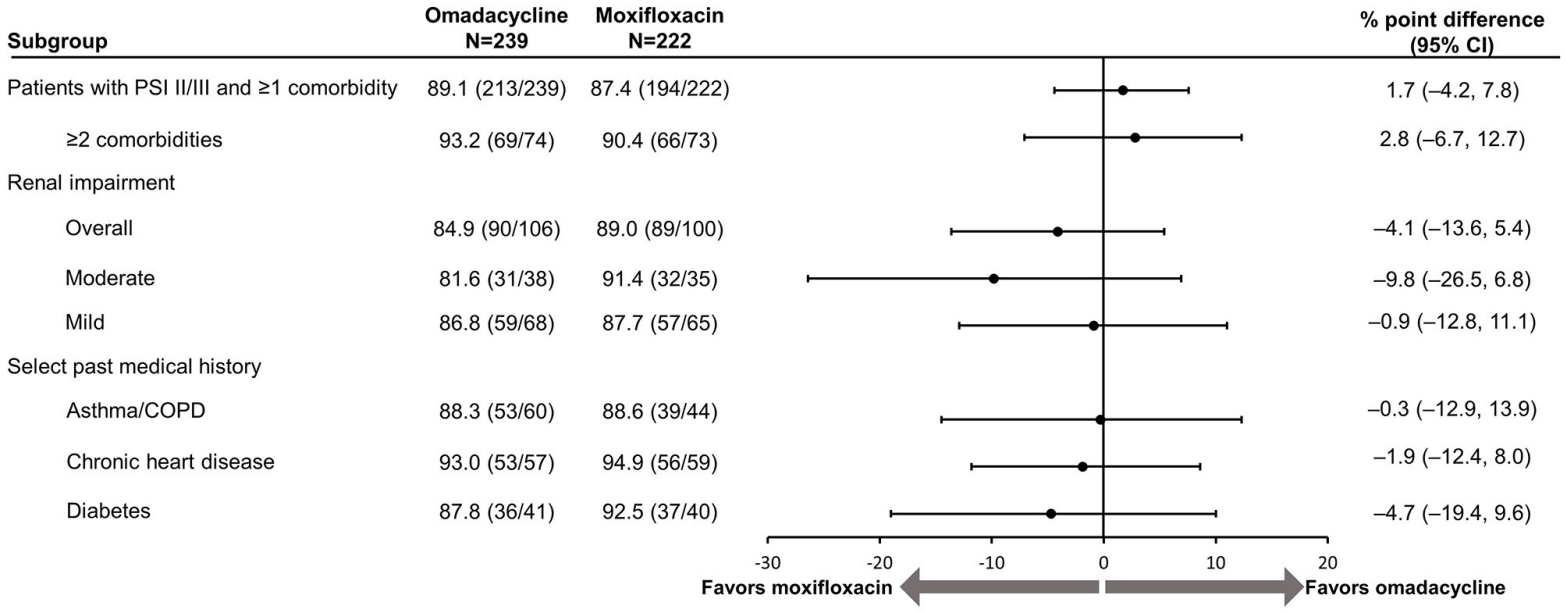
Efficacy of omadacycline and moxifloxacin by comorbidity at post-treatment evaluation, ITT population. CI, confidence interval; COPD, chronic obstructive pulmonary disease; ITT, intention-to-treat; PSI, Pneumonia Severity Index. Renal impairment was defined as creatinine clearance ≤89 mL/min (mild impairment: >60–89 mL/min, moderate impairment: >30–60 mL/min). The 95% CI is based on the Miettinen and Nurminen method without stratification (8). Scores on the PSI are used to place patients with pneumonia into risk classes that range from I to V, with higher risk classes indicating a greater risk of death; in this analysis, only patients in risk classes II (PSI score, 51–70) and III (71–90) were included.

PTE results remained consistent for overall response in both treatment groups (omadacycline: 89.1%; moxifloxacin: 87.4%; Figure 2) and across individual comorbidity subgroups (omadacycline: 84.9% [any renal impairment] to 93.0% [chronic heart disease]; moxifloxacin: 88.6% [asthma/COPD] to 94.9% [chronic heart disease]). There were no significant differences in the PTE efficacy of either omadacycline or moxifloxacin.

### 3.4. Safety

Table 3 shows that treatment-emergent adverse event rates were similar across treatment groups (omadacycline: 38.8%; moxifloxacin: 44.6%), with the most reported adverse events being alanine aminotransferase increase (3.8% vs. 4.1%) and gamma-glutamyltransferase increase (3.8% vs. 2.3%) for omadacycline-treated patients and diarrhea (1.3% vs. 6.3%) for moxifloxacin-treated patients. Discontinuation due to an adverse event was infrequent in both treatment groups (omadacycline: 4.2%; moxifloxacin: 5.9%).

**Table 3:** Treatment-emergent adverse events in patients with Pneumonia Severity Index risk class II/III and ≥1 comorbidity, safety population^a^.

**Preferred term**	**Omadacycline (*N* = 239), *n* (%)**	**Moxifloxacin (*N* = 222), *n* (%)**
Treatment discontinuation for adverse event	10 (4.2)	13 (5.9)
Patients with ≥1 treatment-emergent adverse event	92 (38.8)	99 (44.6)
**Adverse events occurring in >2% of patients in either group^b^**		
Alanine aminotransferase increased	9 (3.8)	9 (4.1)
Gamma-glutamyltransferase increased	9 (3.8)	5 (2.3)
Hypertension	7 (3.0)	4 (1.8)
Headache	6 (2.5)	4 (1.8)
Insomnia	6 (2.5)	3 (1.4)
Aspartate aminotransferase increased	5 (2.1)	7 (3.2)
Constipation	5 (2.1)	1 (0.5)
Nausea	5 (2.1)	8 (3.6)
Vomiting	5 (2.1)	4 (1.8)
Diarrhea	3 (1.3)	14 (6.3)

a The safety population included patients who underwent randomization and received any amount of the trial drug. Adverse events that arose after treatment initiation were those with an onset or worsening of severity that occurred at or after the administration of the first dose of the trial drug until the time of the final follow-up visit.

b *Clostridium difficile* infection (reported as *C. difficile* infection, *C. difficile* colitis, or pseudomembranous colitis) was reported in zero patients in the omadacycline group and in 1 patient (0.4%) in the moxifloxacin group.

## 4. Discussion

This study assessed the safety and clinical efficacy of omadacycline compared with the respiratory fluoroquinolone moxifloxacin for the treatment of adult CABP patients with PSI risk class II/III and at least one comorbidity through a *post-hoc* analysis of the phase 3 OPTIC study. Given their underlying comorbidities, these patients could have been considered eligible for outpatient treatment according to the 2019 ATS/IDSA CAP treatment guidelines (1) and warranted expanded treatment with combination therapy or a respiratory fluoroquinolone monotherapy.

In the OPTIC study, omadacycline was non-inferior to moxifloxacin for the treatment of adult patients with CABP (13). This analysis further supports the results of the OPTIC study, as omadacycline demonstrated similar clinical efficacy to a respiratory fluoroquinolone for the treatment of CABP in adult patients with comorbidities.

Safety concerns have been associated with the fluoroquinolone class since 2008, when the US Food and Drug Administration (FDA) added the first black box warning (15). To date, there are four unique black box warnings and recommendations to limit the use of fluoroquinolones. These include limitations of use for the treatment of acute bacterial sinusitis, acute exacerbations of chronic bronchitis, and uncomplicated urinary tract infections (15). The risks generally outweigh the benefits, as potential serious AEs may be considered worse than the conditions the fluoroquinolones are treating; it is necessary to consider alternative treatment options (15). In response to the FDA warnings, hospitals and antimicrobial stewardship teams have significantly reduced the hospital prescribing of fluoroquinolones; however, most of the antibiotic course is often completed as an outpatient (16), and discharge prescriptions remain unchanged, accounting for the majority of fluoroquinolone use (17).

Fluoroquinolones remain one of the most commonly prescribed antibiotic classes (18–20), likely due to all the reasons cited in the ATS/IDSA guidelines (1), in addition to a lack of awareness of additional options such as omadacycline. The 2019 ATS/IDSA CAP treatment guidelines recommend that patients with any number of comorbidities receive a broader-spectrum treatment due to their increased risk for poor outcomes and risk factors for drug resistance (1). ATS/IDSA guidelines recommend combination therapy to provide the greatest coverage as it is difficult to differentiate between pneumonia caused by typical or atypical pathogens, and patients with comorbidities are at higher risk of severe infection by these pathogens (1). Infection with atypical pathogens, such as *Legionella* spp., may lead to severe pneumonia, and fluoroquinolones can provide broad coverage when used as monotherapy (21). Respiratory fluoroquinolones maintain many advantages over combination therapy; all are once-daily monotherapy, bioequivalent oral therapy with potent *in vitro* activity against the most common cause of bacterial pneumonia, *S. pneumoniae*, and, in addition, have good penetration into the respiratory tissues and fluids (1, 22). All of these attributes, along with CAP being one of the most common indications for antibiotics, result in the unchanged and high utilization of fluoroquinolones for outpatients (17). Despite this, older patients with comorbidities are the group at greatest risk of fluoroquinolone-related adverse events (15).

Outpatients with comorbidities need an efficacious alternative to fluoroquinolones, given the warnings and precautions associated with the antibiotic class. Omadacycline, a derivative of the tetracycline class, has a materially different safety profile from fluoroquinolones, as evident in the warnings and precautions section of the prescribing information (10, 23, 24). While the TEAEs were similar across treatment groups, the study was not powered to detect a difference, and such a study would require a significant increase in enrollment. Given this limitation, post-marketing safety reporting is required by drug manufacturers. In addition, healthcare providers and the public can report via MedWatch, the FDA's medical safety reporting program, which also publishes safety alerts, including those issued for respiratory fluoroquinolones (25). As such, given that omadacycline maintains similar efficacy (13) and the benefits of respiratory fluoroquinolones without the associated black-boxed warnings, it is an important treatment option for patients with CABP, particularly those with comorbidities.

Limitations of this analysis include all limitations inherent to a *post-hoc* study design, such as a restriction of statistical power due to the smaller patient groups following the stratification of the larger cohort into the subgroups assessed. In addition, the patients included in this analysis were considered eligible for outpatient treatment based on objective scoring systems only and did not consider the provider's clinical judgment. Finally, patients may have comorbidities that were excluded from the OPTIC study, specifically other forms of chronic heart, lung, liver, or renal diseases other than those specified herein, and malignancy, alcoholism, and asplenia, which were not captured as comorbidities in OPTIC.

In summary, both omadacycline and moxifloxacin exhibited similar efficacy in patients with PSI risk class II/III and comorbidities. Omadacycline fulfills an unmet need as an oral monotherapy treatment option for adult patients with CABP, which will further reduce the use of fluoroquinolones.

## Data availability statement

The raw data supporting the conclusions of this article will be made available by the authors, without undue reservation.

## Ethics statement

The studies involving human participants were reviewed and approved by the relevant Institutional Review Board or Ethics Committee at each of the 86 participating sites in the OPTIC study. The patients/participants provided their written informed consent to participate in this study.

## Author contributions

All authors contributed to analyzing and interpreting the data, drafting the manuscript, and approving the final manuscript.
